# Analysis of the Influence of Personality Traits on the Level of Knowledge and Health Behaviours of Cardiac Patients

**DOI:** 10.3390/jcm13226856

**Published:** 2024-11-14

**Authors:** Patrycja Krężel, Sylwia Połomska, Anna Jurczak, Grzegorz Czajkowski, Izabela Napieracz-Trzosek, Sylwia Wieder-Huszla

**Affiliations:** 1 Department of Specialized Nursing, Pomeranian Medical University in Szczecin, 71-210 Szczecin, Poland; anna.jurczak@pum.edu.pl (A.J.); izabela.napieracz.trzosek@pum.edu.pl (I.N.-T.); sylwia.wieder.huszla@pum.edu.pl (S.W.-H.); 2 Intensive Cardiac Care Unit, Independent Public Voivodship Hospital, Ul. Arkońska 4, 71-455 Szczecin, Poland; sylwiapolomska1@wp.pl; 3 Independent Unit of Emergency Medical Services, Pomeranian Medical University in Szczecin, 71-210 Szczecin, Poland; grzegorz.czajkowski@pum.edu.pl

**Keywords:** personality, cardiac diseases, health behaviour, knowledge

## Abstract

**Background/Objectives**: Numerous risk factors of cardiac diseases are influenced by health behaviours. An individual’s health behaviours, somatic symptoms and even cardiac outcomes can be influenced by their personality. The aim of this study was to analyse the influence of personality traits on the knowledge and health behaviour of cardiac patients. **Methods**: The study was conducted at the Independent Public Voivodship Hospital in Szczecin on 180 patients in the invasive cardiology wards and the intensive cardiac care unit between July and September 2019. A self-developed questionnaire and the NEO-Five-Factor Inventory-3 (NEO-FFI) and Health Behaviour Inventory (HBI) standardised tools were used. **Results**: Respondents mostly linked the occurrence of cardiovascular disease with smoking (87.22%) and hypertension (85.56%). A large majority of the respondents (68.44%) knew the correct recommended values for blood pressure. This was also the case for total cholesterol—only 20.56% of the patients did not know the correct levels. Respondents experienced more difficulty in adhering to preventive measures—only 27.22% followed a low-fat diet and only half controlled their weight. The overall HBI scale score was 5.93 sten, which indicates an average score. The most intense personality trait among the respondents was extraversion (5.79), while the least intense was agreeableness (4.12). Neuroticism was found to be negatively correlated (*p* < 0.05) with healthy eating habits and a positive mental attitude. Extroverts, on the other hand, are more likely to take preventive action. Older and non-working individuals are more likely to exhibit health-promoting behaviours in all HBI categories (*p* < 0.05). **Conclusions**: The personality traits that have the greatest impact on health behaviour are extraversion, agreeableness and conscientiousness. Health practises also depend on gender, age and work activity. Further research on more diverse groups is needed.

## 1. Introduction

Despite a noticeable decrease in mortality from cardiovascular disease (CVD), it remains the leading cause of death both in Poland and worldwide [1,2]. These are often premature deaths, i.e., deaths that occur before the age of 70 [3]. In Europe, more than 4 million people die annually from CVD, accounting for almost half (45%) of all deaths. Similarly, in Poland, the cardiovascular mortality rate has remained around 45% for many years [4,5].

Cardiovascular disease is associated with both modifiable and non-modifiable risk factors. Non-modifiable factors include age (men over the age of 55 and women over the age of 65), gender (males have an increased risk of developing CVD) and family background (a family history of premature cardiovascular is considered a high-risk factor) [6,7].

Even though non-modifiable factors cannot be changed, the World Health Organization (WHO) states that most cardiovascular disease can be prevented by avoiding behavioural risk factors such as physical inactivity, smoking, excessive alcohol consumption and poor dietary habits [8]. Other modifiable CVD risk factors include obesity, diabetes, hypertension and dyslipidaemia—having either low levels of high-density lipoprotein cholesterol (HDL-C) or high levels of low-density lipoprotein cholesterol (LDL-C) is associated with an increased risk of developing cardiovascular disease [8,9,10,11].

It is evident that there are many factors within an individual’s control that will affect their cardiovascular system in either a positive or negative way. These are so-called health behaviours, i.e., all activities that a person engages in that affect their health in various aspects. There are four groups of health behaviours: those related to physical health, those related to mental health, preventive behaviour and the avoidance of risky activities [12].

Patients’ knowledge of CVD risk factors can influence their health behaviour. However, awareness that certain habits may adversely affect the cardiovascular system does not always correlate with adherence to health-promoting behaviour. Studies report that, although patients are often aware of the risk factors, they still tend to have unhealthy diets or lack physical activity [13]. Studies also confirm that how a person perceives their health is dependent on their character, and the behaviours they will pay the most attention to and implement in their life are related to their personality. Moreover, certain personality characteristics may predispose them to the development of CVD and, in cases of occurrence, influence the outcome [14].

Of the various theories regarding personality types, the theory of the Big Five personality traits is frequently used. It includes neuroticism (anxiousness, hostility and frequent feelings of frustration), extraversion (optimistic attitude, talkativeness and a friendly nature), agreeableness (an ability to cooperate, a willingness to help others and an empathetic and friendly character), conscientiousness (goal-oriented, reliable and responsible) and openness to experience (creativity and a willingness to explore new ideas) [15,16,17]. Many studies report that people with neurotic traits and low conscientiousness have a poorer perception of their health and are more likely to exhibit unhealthy behaviours such as smoking, lack of physical activity or alcohol consumption. On the other hand, neuroticism correlates positively with adherence to taking prescribed medications and recommendations received by medical professionals [17]. High conscientiousness is also a trait that predisposes patients to adhere better to medical regimes, such that they are more likely to implement health-promoting behaviours [18].

Personality types can also be characterised into type A (often associated with workaholism, striving to achieve as many goals as possible as quickly as possible and often displaying aggressive and hostile behaviours), type B (calm, adaptable and communicative, with a tendency to procrastination), type C (characterised by passiveness, introversion, obedience and an ability to cooperate) and type D (pessimistic, anxious and uncertain) [19]. Type A and D are often associated with higher CVD risk, poorer treatment outcomes and higher mortality. In addition, cardiac patients with personality type D are more likely to have a lower quality of life and to experience more symptoms. They are also more likely to exhibit anti-health behaviour, such as tobacco use [16,20]. Therefore, considering a patient’s personality traits can help predict the health behaviours they will exhibit, which may greatly facilitate the educational process through focus on the specific aspects of health-promoting behaviours that the patient has difficulty implementing. This allows for a more holistic therapy to be provided that can contribute to faster therapeutic success. The aim of this study was to analyse the influence of personality traits on the level of knowledge and health behaviours of cardiac patients.

## 2. Materials and Methods

### 2.1. Study Design and Population

A diagnostic survey method was applied using a self-designed questionnaire and standardised tools. The study was conducted from July to September 2019 at the Independent Public Voivodship Hospital in Szczecin, Poland on patients of the invasive cardiology wards and the intensive cardiac care unit. In total, 180 patients were included—103 women and 77 men. The study was conducted in accordance with the Declaration of Helsinki, and the protocol was approved by the Bioethical Commission of Pomeranian Medical University in Szczecin (Approval no. KB-0012/46/01/2013). Participants were informed in advance that the survey was completely anonymous, that completion of the questionnaire was voluntary and that the results would be used for research purposes. Each patient was given written information regarding the purpose and conduct of the study, along with written assurance that they could resign at any stage of the research without having to provide reasons for their decision. The exclusion criteria for the study were an age below 18 years, impaired consciousness and a lack of voluntary consent to participate.

### 2.2. Research Instruments

The study was based on a survey conducted using a questionnaire technique. The following standardised research tools were used to collect empirical data: the NEO Five Factor Inventory (NEO-FFI) by P.T. Costa and R.R. McCrae [21] and the Health Behaviour Inventory (HBI) by Z. Juczyński [22].

We also used an original questionnaire addressing basic sociodemographic data (sex, age, place of residence, employment status, education, marital status and occupational activity), anthropometric data (height, weight and information on forms of physical activity), participants’ knowledge (i.e., regarding the correct norms for blood pressure and cholesterol values, factors affecting cholesterol reduction and risk factors for cardiovascular disease) and the sources of respondents’ knowledge on these topics.

#### 2.2.1. NEO Five Factor Inventory (NEO-FFI)

This tool is used to assess personality traits and is a questionnaire consisting of 60 statements. For each statement, the respondent selects a rating on a five-point scale that best applies to them. These include 1—strongly disagree, 2—disagree, 3—neutral, 4—agree and 5—strongly agree. The NEO-FFI personality questionnaire comprises 5 scales measuring neuroticism, extraversion, openness to experience, agreeableness and conscientiousness. The score from each scale of the Personality Inventory is summed according to the key [21].

#### 2.2.2. Health Behaviour Inventory (HBI)

The Health Behaviour Inventory is a Polish tool designed to measure health-related behaviour in both healthy and sick adults. It contains 24 statements describing health-related habits and categorises individual behaviours into 4 categories, i.e., correct eating habits, preventive behaviour (compliance with recommendations and seeking health-related information), health practises (sleeping habits, physical activity and recreation) and positive mental attitude. The respondent completing the HBI questionnaire marks the frequency of specific activities on a scale from 1 to 5: 1—almost never, 2—rarely, 3—from time to time, 4—often and 5—almost always. When interpreting the results, the obtained scores are summed and range between 24 and 120. The higher the score, the more intensely a person displays the declared behaviours. The number of points is converted into sten, ranging from 1 to 10. A score of 1–4 indicates a low health behaviour, 5–6 represents average health behaviour and 7–10 indicates a high health behaviour [22].

### 2.3. Statistical Analyses

In the study, statistical analysis was performed using the statistical package PQStat version 1.8.0.196. The associations between the NEO-FFI and HBI questionnaires were analysed by estimating Spearman’s rank correlation coefficients. The results of the HBI subscales in relation to gender, marital status, education and occupational activity were compared using the Mann–Whitney test. Associations of age with HBI questionnaire subscales were analysed by estimating Spearman’s rank correlation coefficients. The analysed variables exhibited a non-normal distribution, which dictated the use of non-parametric tests. A test probability of *p* < 0.05 indicates that the results are significant, and a test probability of *p* < 0.01 indicates that they are highly significant.

## 3. Results

The majority of those surveyed were women (57.22%). The majority of respondents (68.89%) were married or cohabiting. Participants most frequently reported having secondary education (35.56%) or higher education (34.44%). Over half of the respondents were retired (51.67%). The mean age was 62.11 (SD—12.74), the mean body weight was 78.27 (SD—17.81) and the mean height was 167.54 (SD—8.93). The body mass index (BMI) was calculated from the collected data and had a mean value of 27.76 (SD—5.40).

### 3.1. Analysis of Health Status and Knowledge About Health Behaviours Among Surveyed Cardiac Patients

**Figure 1:**
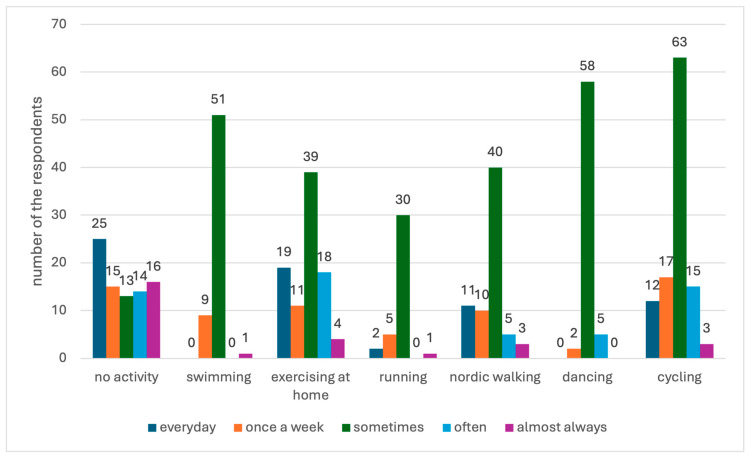
Physical activity and its frequency.

Almost half of the respondents (46%) did not engage in any physical activity (PA). Most of those who did engage in physical activity did so occasionally, with the most common activities being cycling (35%) and dancing (32.22%). Figure 1 shows the numerical distribution of respondents’ answers in terms of undertaken activity and its frequency.

The vast majority of respondents (68.33%) knew the correct recommended values for blood pressure, and almost half (45%) could indicate the correct levels for total cholesterol. Respondents also indicated factors that lower cholesterol levels and most frequently selected a reduction in fat intake (87.78%). Detailed data are shown in Table 1.

**Figure 2:**
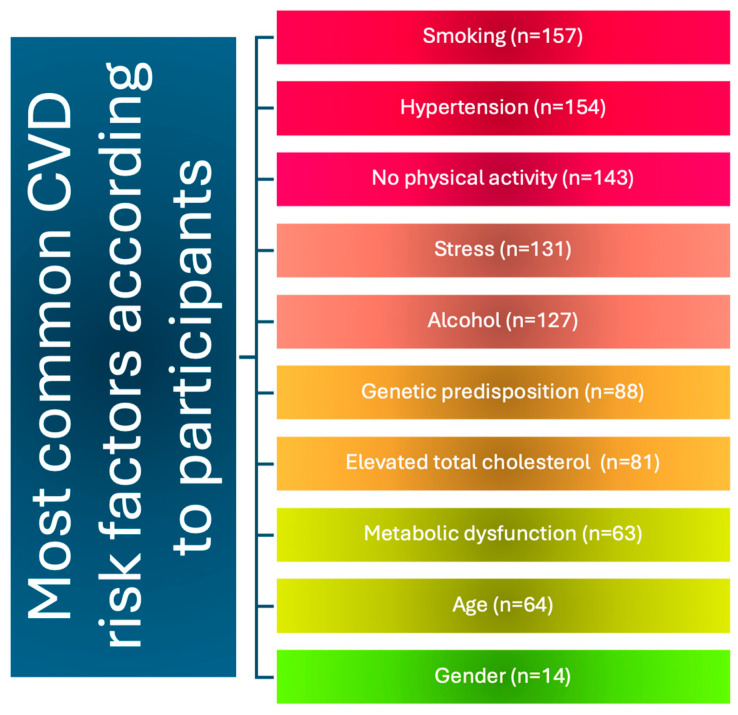
Most common risk factors according to participants.

According to the vast majority of patients, smoking is a major determinant in the development of cardiovascular disease—87.22% (157). Other indicated causes were hypertension, 85.56% (154); lack of exercise, 79% (143); stress, 73% (131); and alcohol, 70.56% (127). Almost half of the respondents (48.89%) indicated that genetic conditions have a strong influence on the development of cardiovascular conditions (Figure 2).

These directly corresponded with the preventative actions taken by participants, with tobacco avoidance (70.56%) considered the most important way to prevent CVD. More than half of the respondents also considered blood pressure (57.78%) and weight control (50%) essential, as well as regular medical visits (51.67%). Respondents mostly sought information about heart disease risk factors from health professionals (43.33%) and the Internet (42.22%) (Table 1).

### 3.2. Analysis Health Behaviour and Personality Traits Among Surveyed Cardiac Patients

**Figure 3:**
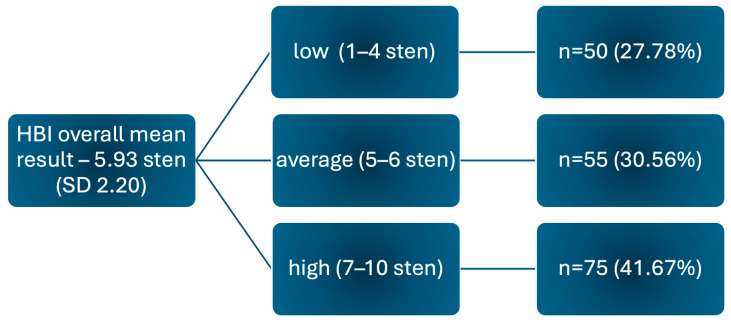
HBI overall health behaviour intensity index.

The overall mean score of HBI scale for the study group is 85.5 (SD 16.59), which is equivalent to 5.93 sten in terms of standardised units and is interpreted as an average result. In terms of the intensity of the overall health behaviour index among respondents, the majority (41.67%) scored at a high level (7–10 sten), as shown in Figure 3.

Analysis of the individual health behaviour categories of the HBI scale showed that the surveyed cardiac patients had the highest mean in the domain of positive mental attitude (21.83 ± 4.58) and the lowest mean in health practises (20.78 ± 4.97). Furthermore, the NEO-FFI questionnaire results revealed that the most intense personality traits in the study group were extraversion (mean 5.79, SD 1.61) and neuroticism (mean 5.3, SD 1.45). The least intense characteristic was agreeableness (mean 4.12, SD 1.42) (Table 2).

Neuroticism is negatively correlated with correct eating habits and a positive mental attitude. Extraversion, in contrast, predisposes patients to better eating habits, preventive behaviours and a positive mental attitude, as indicated by statistically significant correlations in these categories. Similarly, conscientiousness shows a positive correlation with all of the identified health behaviour categories. Furthermore, the analysis shows that women are more likely to implement healthy eating habits. In addition, a highly significant relationship was found between work activity and health practises, with non-workers showing higher levels of health-promoting behaviour in terms of prevention and health practises. Highly significant correlations were also found between age and all areas of the HBI scale, meaning that older patients are more likely to adhere to health-promoting behaviours. The results for the analysis of significant correlations between HBI scale categories and personality traits as well as sociodemographic data (*p* < 0.05) are presented in Table 3 and Table 4.

## 4. Discussion

Cardiovascular disease is a major problem due to its very high mortality rate. Determinants include an unhealthy lifestyle, inability to cope with stress, addictions, lack of physical activity and an inappropriate diet. An individual’s personality may predispose them to the development of somatic disorders, and personality traits may influence the course of pre-existing diseases, exacerbating their symptoms. Addressing personality shortcomings can contribute significantly to the prevention of heart disease and is undoubtedly more economical than treating possible comorbidities. In our study, we aimed to investigate the relationship between personality traits based on the Big Five model and health behaviour according to the HBI scale, which distinguishes multiple specific categories of health behaviour. By doing so, our study was not limited to examining only one factor or only one specific behaviour, thus providing a broader perspective. It appears from our observations that there is a current lack of such studies.

In preventing CVD, knowledge of the causative risk factors is essential. Most patients in this study had satisfactory knowledge regarding blood pressure and total cholesterol norms, as well as the nature of CVD risk factors. However, there is still room for improvement, as patients considered some important factors to be insignificant—such as hyperlipidaemia or age. In addition, 30% of the surveyed patients were smokers, and almost half did not monitor their blood pressure or regularly attend medical appointments. Moreover, only 50% of the patients controlled their body weight as a preventive measure. This shows there is a significant gap between knowing the risk factors and actually implementing health-promoting behaviours. Other studies investigating the knowledge of risk factors among CVD patients found that patients most often considered smoking, lack of PA, hypertension and excessive fat consumption to be important [13,23], although those who declared exercise and a healthy diet to be significant were also more likely to exhibit these behaviours in their daily lives [13]. The only exception were smokers, who continued their harmful habit despite knowledge of the dangers of smoking [13]. The results of numerous studies also illustrate the problem of inactivity among patients with cardiovascular disease. Patients do not exercise at all or have very little physical activity that does not improve their overall health or reduce the risk of mortality [23,24,25,26]. This seems to be a problem worldwide, and the high percentage of inactivity may be due to limitations caused by the presence of CVD, as there are noticeable differences between the physical activity (PA) of cardiac patients and those who have not been diagnosed with cardiovascular disease [23]. The results of our own research confirm this trend, as almost half of the respondents (46%) did not undertake any physical exercise, and those who declared being active most often only occasionally engaged in PA.

As many as 43% of respondents reported that they learned about factors predisposing to cardiovascular disease from healthcare professionals. A study conducted in Lebanon indicated that patients with cardiovascular disease often rely on medical staff as their only source of information [26]. This highlights the important role healthcare professionals have in patient education. Those working in healthcare should emphasise health education on the risk factors of cardiovascular disease and strategies for their reduction to increase patient awareness and thereby lower CVD incidence and mortality [23]. The second most frequent source of information (42%) was the internet. The internet is becoming an increasingly popular, particularly among younger people. A Canadian study found that 25–34-year-olds were the most likely to use the internet to access information about CVD. In contrast, those >55 years of age were more likely to use traditional media. Interestingly, only 20% of all respondents obtained their information from medical professionals [27].

The personality traits of the patients were also analysed. These can impact not only on health behaviour and ways of coping and adapting to living with a CVD but also on predisposition to specific conditions such as hypertension or atherosclerosis. For this reason, certain character traits are considered to be either cardioprotective or predisposing to CVD [28]. In this study, the most intense traits among the patients were extraversion, characterised by high levels of social interaction and more frequent feelings of positive emotions, and neuroticism, namely the tendency to experience negative emotions and heightened sensitivity to stress. Neuroticism has been associated with a higher incidence of CVD, worse disease outcome and higher mortality [29,30,31]. There is also a study indicating that neurotic individuals are more likely to experience adverse morphological changes in the cardiovascular system, including worse left ventricular function and its reduced mass, arterial stiffness and myocardial fibrosis [31]. Similar conclusions were drawn from studies that were based on an assessment of type D personality, which is very similar to the neuroticism in the Big Five model. In addition, it was observed that, although individuals with a type D personality tend to have more severe symptoms, they are less likely to report them and seek medical attention. They also often have problems adapting to a new situation, e.g., after interventional cardiac treatment, and are more likely to exhibit depressive symptoms [32], which correspond with our own findings—that neuroticism is correlated with more negative mental attitude and poor eating habits. This supports the notion that neuroticism has a negative impact on health behaviours. As significant correlations were only found in these two categories of the HBI scale, it may be concluded that these aspects should be the point of focus when working with patients with neurotic traits.

Extraversion, on the other hand, is associated with both negative and positive behaviours. Negative examples mainly include more frequent use of substances, especially cigarettes, which significantly increases the risk of CVD. Similar tendencies have been seen for type A behaviour, as well as minimising the severity of one’s symptoms [29,32]. Positive extrovert behaviour, on the other hand, includes a willingness both to cooperate and to follow medical advice. Extroverts also often have lower blood pressure values and are less prone to hypertension than introverts, which can be due to the higher anxiety and stress levels in introverts [33]. In our study, we found extraversion to be rather advantageous trait. It is positively linked to almost all categories of HBI scale, which means that extroverts have a better mental attitude, higher engagement in preventive behaviour and are more likely to have a healthy diet.

In this study, conscientiousness exhibited positive correlations (*p* < 0.05) with all categories of health behaviours of the HBI scale. It is the only trait that has associations with all HBI scale categories and with such highly significant correlations. According to many studies, conscientiousness is the trait that most predisposes to health-promoting behaviours such as undertaking PA, maintaining a healthy diet, taking medication as prescribed, avoiding tobacco and alcohol or willingness to comply with medical recommendations. This means people who are highly conscientious are less likely to develop chronic diseases, including cardiovascular disease, often have a milder disease course and even have a lower risk of death. Conscientiousness is even described as a cardioprotective trait in some studies [14,18,29,34]. Therefore, the highly significant impact of conscientiousness on health-promoting behaviour is confirmed by our study.

Other fairly positive qualities, though less prominent than conscientiousness, are agreeableness and openness to experience. It has been found that higher openness is associated with better physical condition and more frequent physical activity [14]. However, openness to trying new things also manifests as more frequent use of alternative medicine and is associated with poorer compliance with medical recommendations [18]. Nonetheless, it is also regarded as a cardioprotective trait, and individuals who exhibit openness are less likely to develop coronary heart disease and are at lower risk of dying from cardiac causes [35]. In the current study, no correlation was found between openness to experience and health behaviours. This may be related to the studied group being insufficiently differentiated and the research conducted in a single centre, which highlights the need for further research. Furthermore, agreeableness also was not correlated with any of the health behaviours and was the least intense trait among the respondents. This may be due to the older age of the participants, as other studies indicate that they are less likely to exhibit agreeableness in comparison with younger patients. In addition, people with heart failure and coronary heart disease tend to be less agreeable [18].

In this study, factors such as gender, age and work activity were found to be associated with health behaviour. Almost 58% of the respondents were women. Our study showed that women are more likely to have good eating habits, though the correlation is weak. However, other studies show that women tend to consume less meat and high-fat products, which may be related to being more mindful of their diet, among other reasons [36]. Still, we determined an average BMI of 27.76, a result that corresponds to being overweight. This may be related to the fact that women are less likely to engage in physical activity than men [37,38]. It has also been shown that women tend to eat more in situations of negative emotions and stress. This type of behaviour may contribute to being overweight [36]. In addition, women are more likely to be neurotic and agreeable than men, and neuroticism is often associated with a lower level of activity [39].

Furthermore, we observed some association of age and work activity to health behaviours—older and non-working individuals are more likely to take better care of their health and maintain a healthy lifestyle. This is in accordance with the results of other studies, which show that health-promoting behaviour is more frequently demonstrated with increasing age. Older patients are more likely to take their medication as prescribed and are less likely to exhibit harmful habits such as smoking [40,41]. In addition, older people tend to exhibit fewer neurotic behaviours [18]. Thus, the fact that older people have better health practises than younger people may be due to a lower level of neuroticism and simply the fact that they have more time to improve their knowledge and take care of their health. The latter also applies to non-working people.

No correlation was found between education and health behaviour. However, research indicates that individuals with higher education possess greater knowledge about health-related behaviours and have higher expectations regarding the healthcare they receive. People with higher education are also more concerned with how to reduce stress and effectively deal with difficult situations [42,43].

This study has some limitations. First, it was conducted in a single hospital and the surveyed patients had a high mean age, which may restrict the generalisability of the results. Furthermore, the cross-sectional nature of the study may limit the possibility of making inferences about causality between personality traits and health behaviour. Undoubtedly, conducting research on a larger population in several healthcare facilities with a wider age range could add value to the current study. However, our study is only preliminary, and further research on such comparisons is warranted.

## 5. Conclusions

Due to the seriousness of their condition, patients with cardiovascular disease often seek information about their illness and its associated risk factors. They do not always research the subject sufficiently and have some gaps in their knowledge. However, they try to follow the available recommendations and avoid the most common risk factors. It is also common for patients to seek information from healthcare workers. Medical professionals are perceived as reliable sources of information and can easily interact with patients, assessing their knowledge and personality while supporting them in taking care of their own health and preventing the development of further cardiac conditions or the worsening of existing symptoms. The results also demonstrate that personality traits impact on patients’ health behaviour, not only generally but also in specific categories. This is only a preliminary study, and its limitations must be considered. However, the results still suggest that while planning a patient’s care and education, it is worthwhile to first identify and assess their predominant personality traits, as this can help to determine the probability that specific health behaviours will be present, which can be managed in cooperation with the patient—correct habits can be encouraged and unhealthy tendencies corrected. It might also help in assessing what behaviours can be expected and what possible difficulties may arise during the educational process. In future, larger-scale research with a more diverse sample group and extensive statistical analysis is needed.

## Figures and Tables

**Table 1:** Knowledge of surveyed patients on blood pressure norms, cholesterol levels and factors lowering cholesterol levels, as indicated by the participants.

Variables	*n*	%
Blood pressure norms according to the respondents		
Lower than 120/80 mmHg	123	68.33
Around 140/90	54	30.0
Around 170/100	0	0.00
Around 100/60	3	1.67
Correct total cholesterol levels according to respondents		
Below 200 mg/dL	81	45.0
Above 300 mg/dL	3	1.67
Around 150 mg/dL	59	32.78
I don’t know	37	20.56
Factors lowering cholesterol levels according to the respondents		
Limiting carbohydrates	66	36.67
Body weight reduction	112	62.22
Reduction in fat intake	158	87.78
Reduction of exertion	9	5.00
Preventative actions taken by the participants		
Weight control	90	50
Avoidance of smoking	127	70.56
Low-fat diet	49	27.22
Blood pressure control	104	57.78
Regular medical appointments	93	51.67
Source of information		
Journals and books	70	38.89
Internet	76	42.22
Friends and family	27	15
Television and radio broadcasts	62	34.44
Medical professionals	78	43.33

**Table 2:** HBI scale categories and NEO-FFI questionnaire results.

Variables	Mean	SD
HBI categories		
Correct eating habits	21.48	4.69
Preventive behaviour	21.41	4.95
Positive mental attitude	21.83	4.58
Health practises	20.78	4.97
NEO-FFI personality traits		
Neuroticism	5.3	1.45
Extraversion	5.79	1.61
Openness to experience	5.23	1.86
Agreeableness	4.12	1.42
Conscientiousness	5.12	1.55

SD—standard deviation.

**Table 3:** Relations of HBI scale categories to personality traits and age.

Variables		Correct Eating Habits	Preventive Behaviour	Positive Mental Attitude	Health Practises
Neuroticism	R	−0.181	−0.114	−0.196	−0.097
	*p*	0.015	0.127	0.008	0.194
Extraversion	R	0.144	0.184	0.215	0.013
	*p*	0.053	0.013	0.004	0.865
Openness to experience	R	−0.046	−0.045	0.021	−0.134
	*p*	0.537	0.549	0.775	0.072
Agreeableness	R	0.109	0.101	0.143	0.089
	*p*	0.146	0.179	0.055	0.232
Conscientiousness	R	0.195	0.290	0.238	0.164
	*p*	0.009	0.001	0.001	0.028
Age	R	0.194	0.230	0.175	0.324
	*p*	0.046	0.002	0.019	0.001

*p* < 0.05, R—Spearman’s correlation coefficient.

**Table 4:** Significant relations of HBI scale categories to occupational activity and gender.

HBI Categories	Occupational Activity	M ± SD	z	*p*
Correct eating habits	Active	20.58 ± 4.08	2.327	0.0200
	Inactive	22.06 ± 4.97		
Preventive behaviour	Active	20.18 ± 4.60	2.869	0.0041
	Inactive	22.21 ± 5.03		
Positive mental attitude	Active	20.90 ± 4.22	2.354	0.0186
	Inactive	22.43 ± 4.72		
Health practises	Active	18.80 ± 4.21	4.522	0.0001
	Inactive	6.42 ± 2.23		
	**Gender**			
Correct eating habits	Male	20.56 ± 5.21	2.189	0.0285
	Female	22.16 ± 4.14		

M—mean, SD—standard deviation, z—z-score, *p* < 0.05.

## Data Availability

The original contributions presented in the study are included in the article, and further inquiries can be directed to the corresponding authors.
